# Retinoic acid-related orphan receptors α and γ: key regulators of lipid/glucose metabolism, inflammation, and insulin sensitivity

**DOI:** 10.3389/fendo.2013.00001

**Published:** 2013-01-25

**Authors:** Anton M. Jetten, Hong Soon Kang, Yukimasa Takeda

**Affiliations:** Cell Biology Section, Division of Intramural Research, National Institute of Environmental Health Sciences, National Institutes of HealthResearch Triangle Park, NC, USA

**Keywords:** retinoic acid-related orphan receptor, obesity, inflammation, adipose tissue, hepatosteatosis, diabetes, insulin-resistance, circadian rhythm

## Abstract

Retinoic acid-related orphan receptors RORα and RORγ play a regulatory role in lipid/glucose homeostasis and various immune functions, and have been implicated in metabolic syndrome and several inflammatory diseases. RORα-deficient mice are protected against age- and diet-induced obesity, hepatosteatosis, and insulin resistance. The resistance to hepatosteatosis in RORα-deficient mice is related to the reduced expression of several genes regulating lipid synthesis, transport, and storage. Adipose tissue-associated inflammation, which plays a critical role in the development of insulin resistance, is considerably diminished in RORα-deficient mice as indicated by the reduced infiltration of M1 macrophages and decreased expression of many proinflammatory genes. Deficiency in RORγ also protects against diet-induced insulin resistance by a mechanism that appears different from that in RORα deficiency. Recent studies indicated that RORs provide an important link between the circadian clock machinery and its regulation of metabolic genes and metabolic syndrome. As ligand-dependent transcription factors, RORs may provide novel therapeutic targets in the management of obesity and associated metabolic diseases, including hepatosteatosis, adipose tissue-associated inflammation, and insulin resistance.

## INTRODUCTION

In the past 50 years, the occurrence of obesity has greatly increased worldwide in both adults and children and has become a major health-care concern in many countries. In the United States 30% of the population is considered obese, while more than 66% of adults and almost 17% of children and adolescents are overweight (Browning et al., 2004; Ogden et al., 2012). Obesity is associated with an increased risk of several pathologies, including type 2 diabetes, cardiovascular disease, and non-alcoholic fatty liver disease (NAFLD). Accumulating evidence indicates that networks regulating lipid metabolism and inflammation are highly integrated and play a critical role in the development of these pathologies (Hotamisligil, 2006; Donath and Shoelson, 2011; Ouchi et al., 2011; Glass and Olefsky, 2012). Obesity leads to a systemic state of low-grade inflammation, particularly involving adipose tissue, that is causally involved in the development of insulin resistance and other diseases. Blood levels of free fatty acids (FFA) are elevated in obesity and through their interaction with Toll-like receptor 4 (TLR4) FFA induce proinflammatory pathways in macrophages and other cell types that may promote insulin resistance (Samuel and Shulman, 2012). Recent studies demonstrated that retinoic acid-related orphan receptors (RORs) are among many factors that through their modulation of immune responses and lipid/glucose homeostasis regulate the development of inflammation, metabolic syndrome, and insulin resistance (Jetten, 2009; Solt and Burris, 2012).

## RORα AND γ PROTEINS

The RORs alpha, beta, and gamma (RORα–γ or NR1F1–3) constitute a subfamily of nuclear receptors that function as ligand-dependent transcription factors (Jetten, 2004, 2009; Solt and Burris, 2012). RORs exhibit a domain structure typical of nuclear receptors and contain an N-terminal domain, the function of which has not yet been clearly defined, a highly conserved DNA-binding domain (DBD) consisting of two zinc finger motifs, a LBD, and a hinge domain spacing the DBD and LBD. By using different promoters and/or alternative splicing each ROR gene produces several isoforms that vary only in their N-terminal region. Some of these isoforms exhibit a distinct tissue-specific pattern of expression and control different genes and biological processes. RORs regulate transcription by binding as monomers to ROR response elements (RORE), which consist of the core sequence “AGGTCA” preceded by an A/T-rich sequence, in the regulatory region of target genes. The activation function (AF-2), localized at the C-terminus within the LBD of RORs, is involved in the recruitment of co-activators or co-repressors that mediate the transcriptional activation or repression by RORs. Recent studies have identified a number of (ant)agonists that interact with the LBD of ROR and either activate or inhibit ROR transcriptional activity (Kallen et al., 2002; Huh and Littman, 2012; Solt and Burris, 2012). Interaction with agonists induces a conformational change in the LBD that allows release of the co-repressor complex and promotes assembly of a co-activator complex that mediates the transcriptional activation by ROR, while the inverse happens for antagonists. These observations not only indicated that RORs function as ligand-dependent transcription factors, but also suggested that RORs might be potential therapeutic targets to treat disease.

## RORs AS REGULATORS OF SEVERAL IMMUNE PROCESSES

RORα and RORγ are important regulators of several diverse immune functions. RORγ-deficient mice lack lymph nodes and Peyer’s patches indicating that it is essential for lymph node development (Kurebayashi et al., 2000; Sun et al., 2000). Recent studies demonstrated that RORα and the RORγt isoform play a key role in T cell lineage determination (Ivanov et al., 2006; Yang et al., 2008; Jetten, 2009). The RORγt isoform in particular and to a lesser extent RORα, is required for the differentiation of naïve T cells into interleukin 17 (IL-17) producing T helper 17 (Th17) cells. IL-17A expression is directly regulated by RORs through their interaction with ROREs in the *Il17* promoter (Yang et al., 2008). Proinflammatory Th17 cells and IL-17 have been implicated in several autoimmune diseases and other inflammatory disorders. Deficiency in RORγt or both RORα/γ receptors has been shown to greatly inhibit the generation of Th17 cells and the development of experimental encephalomyelitis in mice. In addition, mice deficient in RORα or RORγ displayed a diminished susceptibility to allergen-induced lung inflammation and collagen-induced arthritis (Jaradat et al., 2006; Tilley et al., 2007) and polymorphisms in RORα have been associated with increased susceptibility to asthma (Ramasamy et al., 2012). A recent study identified a role for RORα in the generation of natural helper (NH) cells (Halim et al., 2012). RORα-deficient, but not RORγ-deficient, mice lack NH cells. NH cell-deficient mice generated by RORα-deficient bone marrow transplantation exhibited normal Th2 cell responses, but failed to develop acute lung inflammation in response to a protease allergen. These findings might at least in part explain the reduced susceptibility to allergen-induced lung inflammation in RORα-deficient mice (Jaradat et al., 2006).

An increased Th17 response has been reported to correlate with white adipose tissue (WAT)-associated inflammation and the development of insulin resistance in obese mice and patients (Ahmed and Gaffen, 2010; Bertola et al., 2012). Whether inhibition of Th17 differentiation plays a role in the protection RORα- and RORγ-deficient mice against diet-induced insulin resistance needs further study. RORα or RORγ have also been implicated in the regulation of thymopoiesis. Loss of RORγt results in accelerated apoptosis of double-positive thymocytes, while RORα deficiency significantly reduces the generation of single positive thymocytes ( Kurebayashi et al., 2000; Sun et al., 2000; Dzhagalov et al., 2004).

## RORα IN DIET- AND AGE-INDUCED OBESITY

Study of *Staggerer* (*RORα^sg/sg^*) mice, a natural mutant strain containing a deletion in the *RORα* gene that results in loss of RORα expression, indicated that RORα plays a critical role in the control of lipid metabolism and the development of various aspects of metabolic syndrome. These investigations showed that *RORα^sg/sg^* mice are protected against age- and diet-induced obesity and the development of several obesity-linked pathologies, including adipose tissue-associated inflammation, hepatosteatosis, and insulin resistance (Kang et al., 2011; Lau et al., 2011). *RORα^sg/sg^* mice fed a high fat diet (HFD) gain relatively less weight and exhibit a significantly lower total body fat index compared to wild-type (WT) littermates on a HFD. Similarly, male *RORα^sg/sg^* mice were also protected against age-induced obesity. Adipose tissue is the main site of storage of excess energy that is stored in the form of triglycerides in single large lipid droplets. The reduced adiposity in *RORα^sg/sg^* mice was largely related to smaller adipocyte size due to diminished deposition of triglycerides.

RORα, particularly the RORα4 isoform, has been shown to be highly expressed in WAT and to be induced during differentiation of D1 and 3T3-L1 preadipocytes (Austin et al., 1998). Overexpression of RORα in preadipocytes inhibits adipocyte differentiation (Duez et al., 2009; Ohoka et al., 2009). This appears to be mediated through a direct interaction of RORα with CCAAT/enhancer-binding protein β (C/EBPβ) that results in the inhibition of the recruitment of the co-activator CBP and repression of C/EBPβ transcriptional activity. These studies suggest that RORα has a negative regulatory role in adipocyte differentiation. This function, however, does not explain the reduced adiposity observed in RORα-deficient mice.

Obesity is a consequence of an imbalance between energy intake and expenditure (Glass and Olefsky, 2012; Samuel and Shulman, 2012). However, the decrease in diet-induced adiposity in *RORα^sg/sg^* mice was found not to be due to reduced food intake or increased fecal lipid excretion. Indirect calorimetric analysis showed that VO_2_, VCO_2_, and heat generation were significantly enhanced in *RORα^sg/sg^* mice on a HFD (Kang et al., 2011). This suggested that elevated energy expenditure might at least in part be responsible for the reduced weight gain and resistance to hepatosteatosis and insulin insensitivity in *RORα^sg/sg^* mice.

## RORα AND WAT-ASSOCIATED INFLAMMATION

**FIGURE 1:**
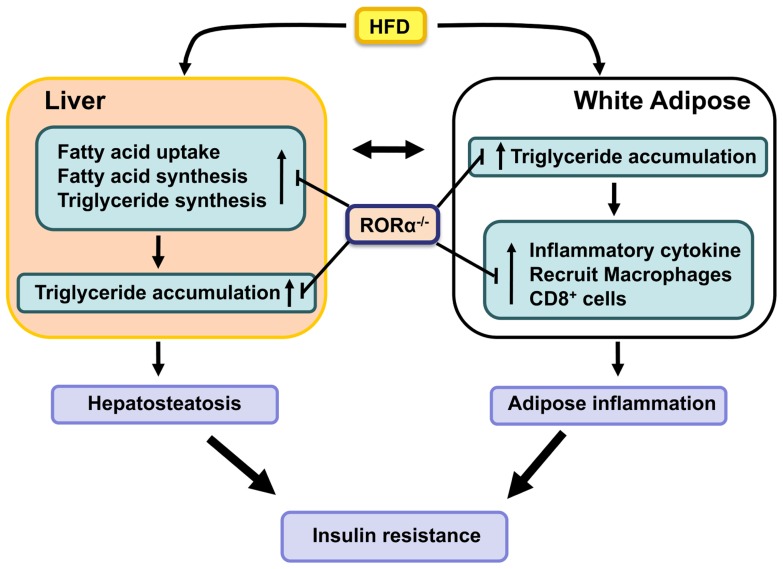
**RORα functions as a positive regulator of hepatosteatosis, WAT-associated inflammation, and insulin-resistance in diet- and age-induced obesity.** Loss of RORα inhibits the hepatic expression of lipogenic genes and suppresses the expression of proinflammatory genes and the infiltration of macrophages in WAT, and as a result protects against these pathologies.

In addition to functioning as the main site of storage of extra energy in the form of triglycerides derived from food intake, white adipocytes produce a variety of endocrine hormones, including leptin, adiponectin, resistin, and retinol-binding protein 4 (RBP-4) which regulate food intake, lipid metabolism, and inflammation (Hotamisligil, 2006; Guilherme et al., 2008; Glass and Olefsky, 2012). Leptin and adiponectin promote insulin sensitivity, while resistin and RBP4 have the opposite effect and impair insulin sensitivity. It is now well-recognized that obesity is associated with a chronic state of low grade, systemic inflammation and that this is an important contributory factor in the development of insulin resistance (Hotamisligil, 2006; Odegaard and Chawla, 2008; Nishimura et al., 2009; Glass and Olefsky, 2012). Progressive infiltration of various immune cells, including macrophages and CD8^+^ effector T lymphocytes, in WAT lead to increased release of proinflammatory cyto- and chemokines. In addition to the accumulation of bone marrow-derived macrophages, there is also a shift from anti-inflammatory “alternatively activated” (CD11c^-^CD206^+^) M2 macrophages to proinflammatory “classically activated” (CD11c^+^CD206^-^) M1 macrophages (Sun et al., 2011; Glass and Olefsky, 2012), which in advanced obesity aggregate into crown-like structures (CLS) surrounding necrotic adipocytes. Recent studies indicated that CD8^+^ T cells are critical in promoting recruitment of macrophages in WAT in obesity (Weisberg et al., 2003; Odegaard and Chawla, 2008; Nishimura et al., 2009). In addition, a reduction in anti-inflammatory T regulatory (Treg) cells and an increase in proinflammatory Th17 response further stimulate WAT-associated inflammation (**Figure 1**).

Deficiency of RORα greatly inhibits diet-induced adipose tissue-associated inflammation in mice (Kang et al., 2011; Lau et al., 2011). This is indicated by the greatly reduced infiltration of macrophages and CD8^+^ T lymphocytes in WAT of *RORα^sg/sg^* mice fed a HFD. This was further supported by the significant reduction in the formation of CLS and the expression of several macrophage markers, such as F4/80, Mac-2, macrophage expressed 1 (Mpeg1), and macrophage scavenger receptor 1 (Msr1), in WAT of *RORα^sg/sg^* mice. Moreover, the relative percentage of proinflammatory M1 macrophages was significantly diminished in *RORα^sg/sg^* WAT. This was supported by flow cytometric analysis and the much lower levels of Cd11c expression. The reduced inflammation in *RORα^sg/sg^* WAT is further indicated by gene expression profiling showing a greatly reduced expression of a large number of chemokines, including *Ccl2*, *Ccl8*, *Ccl3*, and *Ccl7*, the chemokine receptors *Ccr3*, *Ccr5*, and *Ccr7*, the proinflammatory cytokines *Tnfα* and *IL-6*, the interleukin 1 receptor antagonist (*Il1rn*), osteopontin (*Opn*), *CD44*, serum amyloid 3 (*Saa3*), and several TLRs and metalloproteinases in WAT of *RORα^sg/sg^* mice compared to their WT counterparts (Kang et al., 2011). The expression of these genes has been reported to be elevated in obesity and many of these genes have been implicated in obesity-induced inflammation in WAT as well as insulin resistance. For example, both the CCL2/CCR2 and CCL3/CCR5 pathways have been reported to promote recruitment of macrophages in adipose tissue (Kanda et al., 2006; Kitade et al., 2012). CD44, a multifunctional cell membrane protein that acts as a receptor for hyaluronan and Opn, has been shown to regulate migration of macrophages and neutrophils (Johnson and Ruffell, 2009). CD44 and Opn null mice are protected against the development of HFD-induced hepatosteatosis, WAT-associated inflammation, and insulin resistance (Nomiyama et al., 2007; Bertola et al., 2009; Kiefer et al., 2011; Kodama et al., 2012). These observations suggest that suppression of several proinflammatory genes and pathways in *RORα^sg/sg^* WAT is causally linked to the reduced inflammation (**Figure 1**). Future studies have to determine what the primary effects are by which RORα regulate the expression of these genes.

## RORα AND HEPATOSTEATOSIS

Obesity is associated with increased prevalence of NAFLD, which is characterized by elevated lipid accumulation in hepatocytes (Fabbrini et al., 2010). NAFLD develops when the rate of fatty acid uptake and synthesis and subsequent esterification to triglycerides is greater than the rate of fatty acid oxidation and secretion. Advanced NAFLD progresses into increased inflammation and hepatotoxicity. Several studies showed that compared to WT mice hepatic triglyceride levels are considerably reduced in *RORα^sg/sg^* mice fed a HFD or aging male *RORα^sg/sg^* mice (Raspe et al., 2001; Lau et al., 2008; Kang et al., 2011). These observations indicated that *RORα^sg/sg^* mice are protected against the development of age- and diet-induced hepatosteatosis. Gene expression profiling revealed that the expression of a number of lipogenic genes was significantly reduced in the liver of *RORα^sg/sg^* mice fed a HFD. Expression of *Srebp-1c* and fatty acid synthase (*Fas*), key regulators for lipogenesis, was reduced in liver of *RORα^sg/sg^* mice. In addition, the expression of several genes involved in the main pathway of triglyceride synthesis, including glycerol-3-phosphate acyltransferase (*Gpam* or *Gpat1*) and acyl-glycerol-3-phosphate acyltransferase 9 (*Agpat9*) and *Mogat1*, which is part of an alternative pathway of triglyceride synthesis, were significantly diminished in *RORα^sg/sg^* liver (Kang et al., 2011). The hepatic expression of the cell death-inducing DFFA-like effectors a and c (*Cidea* and *Cidec*) and perilipin 2 (*Plin2* or *Adfp*), which play a critical role in the regulation of lipid storage, lipid droplet formation, and lipolysis (Gong et al., 2009; Greenberg et al., 2011), was also suppressed in *RORα*-deficient mice. RORα has been reported to activate *Plin2* transcription directly through interaction with ROREs in the *Plin2* promoter (Kang et al., 2011). Recently, the expression of fibroblast growth factor (*Fgf21*), an important regulator of lipid/glucose metabolism, was found to be directly regulated by RORα in hepatocytes (Wang et al., 2010c). Together these observations suggest that the protection against hepatosteatosis in *RORα^sg/sg^* mice is related to reduced expression of many genes involved in promoting lipogenesis and triglyceride storage, some of which are directly regulated by RORα (**Figure 1**).

## RORα AND INSULIN RESISTANCE

Both adipose-associated inflammation and hepatosteatosis have been linked to the pathogenesis of insulin resistance in obesity (Guilherme et al., 2008; Donath and Shoelson, 2011; Samuel and Shulman, 2012), although a cause-effect relationship not always exists between hepatosteatosis and diabetes (Sun and Lazar, 2013). The phenotypic differences observed between WT and *RORα^sg/sg^* mice fed a HFD are consistent with this correlation. *RORα^sg/sg^* mice, which are protected against obesity, hepatosteatosis, and WAT-associated inflammation, exhibited a significantly reduced susceptibility to diet-induced insulin resistance and glucose intolerance compared to obese WT mice (Lau et al., 2008; Kang et al., 2011). In humans, two studies have revealed a connection between RORα, obesity, and type 2 diabetes. A rearrangement resulting in disruption of human RORα1 was found to be associated with severe obesity (Klar et al., 2005), while a recent GWAS study showed an association between a single nucleotide polymorphism in RORα (rs7164773) and increased risk for type 2 diabetes in the Mexico Mestizo population (Gamboa-Melendez et al., 2012).

Many inflammatory and lipogenic genes, including *Plin2*, *Il1rn*, *Opn*, *CD44*, and *Cidec*, that are down-regulated in *RORα^sg/sg^* mice have been reported to also regulate insulin sensitivity. *Plin2* null mice displayed reduced hepatic lipid accumulation and improved insulin sensitivity and glucose tolerance in an ob/ob background (Chang et al., 2010). *Il1rn*, one of the genes most dramatically repressed in WAT of *RORα^sg/sg^* mice (Kang et al., 2011), has been reported to be highly up-regulated in WAT of obese humans and to regulate insulin sensitivity (Juge-Aubry et al., 2003; Somm et al., 2006). Similarly, *Opn* expression was found to be elevated in obesity, while Opn deficiency was shown to inhibit obesity-induced inflammation and insulin resistance (Bertola et al., 2009; Kiefer et al., 2011). Deficiency in CD44, a receptor for Opn, also results in improved insulin sensitivity (Kodama et al., 2012) suggesting a role for the Opn/CD44 pathway in the control of insulin sensitivity. Mice deficient in Cidea or Cidec, which play a role in lipid storage, are protected from diet-induced obesity and display improved insulin sensitivity (Gong et al., 2009). Thus, the down-regulation of several genes, including *Plin2*, *Il1rn*, *Opn*, *CD44*, and *Cidec* in *RORα^sg/sg^* mice may collaboratively be responsible for the improved insulin sensitivity through their interrelated effects on inflammation, adipogenesis, and lipid homeostasis (**Figure 1**).

In addition to adipose tissue and liver, the pancreas and the skeletal muscle also play important roles in energy homeostasis and insulin resistance. The pancreatic islets produce a number of hormones, including insulin and glucagon, that are critical in the regulation of lipid and glucose homeostasis (Saltiel and Kahn, 2001; Cryer, 2012). RORα was shown to be selectively expressed in the glucagon-producing alpha cells; however, its role in these cells and its relationship to the phenotype observed in RORα-deficient mice needs yet to be established (Mühlbauer et al., 2013). In skeletal muscle, RORα has been reported to regulate the expression of a number of genes involved in lipid and carbohydrate metabolism (Lau et al., 2011). Ectopic expression of an RORα mutant in skeletal muscle C2C12 cells reduced the expression of the lipogenic genes, sterol regulatory element-binding transcription factor 1 (Srebp1), Fas, and stearoyl-CoA desaturase 1 (Scd1), and genes involved in cholesterol efflux, such as ATP-binding cassette, sub-family A, member 1 (Abca1). Caveolin-3 (Cav3) and carnitine palmitoyltransferase-1 (Cpt1) were found to be directly regulated by RORα. Changes in the expression of these genes may be in part responsible for the modulation of lipid and glucose homeostasis by RORα.

In muscle, insulin stimulates glucose uptake by stimulating the translocation of Glut4 (Slc2a4) to the plasma membrane (Rose and Richter, 2005). This involves phosphorylation of the insulin receptor substrate 1 (IRS1), which leads to the activation of phosphatidylinositol 3-kinase (PI3K) and subsequently AKT, which then promotes Glut4 translocation. Recently, evidence was provided for a role of RORα in PI3K-Akt signaling (Lau et al., 2011). Akt1/2 expression was up-regulated in skeletal muscle of *RORα^sg/sg^* mice and this correlated with an increase in the level of insulin-induced Akt phosphorylation, Glut4 expression, and glucose uptake. This stimulation in Akt signaling might at least in part account for the improved insulin sensitivity observed in *RORα^sg/sg^* mice.

## RORγ1 AND INSULIN SENSITIVITY

The RORγ gene generates two different isoforms, RORγ1 and RORγt (RORγ2), that are expressed in a highly tissue-specific manner (Jetten, 2009). Expression of the RORγ1 isoform is restricted to several peripheral tissues, including liver, adipose tissue, kidney, small intestines, pancreas, and skeletal muscle. Recent studies identified RORγ1 as a negative regulator of adipocyte differentiation and a modulator of obesity-associated insulin resistance (Meissburger et al., 2011; Tinahones et al., 2012). In obese *RORγ *^-/-^ mice, the number of adipocytes was increased (hyperplasia), while adipocyte size was reduced. Fasting blood insulin levels were shown to be significantly lower in diet-induced obese *RORγ*^-/-^ mice and in *RORγ*^-/-^ob/ob double knockout mice and mice displayed improved insulin sensitivity. In addition, *RORα*^-/-^ adipocytes were highly insulin sensitive leading to improved control of circulating FFA. These observations are consistent with a recent study showing that, opposed to adipose hypertrophy, obese patients with adipose tissue hyperplasia (many small adipocytes) exhibit better glucose and lipid profiles and might be less susceptible to developing insulin resistance (Hoffstedt et al., 2010) and with data showing that in human patients the level of *RORγ1* expression positively correlated with adipocyte size and insulin resistance (Meissburger et al., 2011; Tinahones et al., 2012). Up to now, no association has been established between *RORγ* polymorphisms and susceptibility to insulin resistance in humans. However, in cattle, a single polynucleotide polymorphism in RORγ has been linked to increased adiposity (Barendse et al., 2007). These observations suggest that the loss or potentially the inhibition of RORγ1 might protect against insulin resistance and type 2 diabetes.

In addition to adipose tissue, regulation of lipid and glucose metabolism in other tissues, including liver, pancreas, and skeletal muscle might be part of the mechanism by which by RORγ modulates insulin sensitivity. In skeletal muscle, RORγ has been reported to regulate the expression of genes associated with lipid and carbohydrate metabolism as well as the production of reactive oxygen species (Raichur et al., 2007). A recent study revealed that RORγ was selectively expressed in insulin-producing pancreatic β cells; however, its role in β cells and how this relates to the modulation of insulin sensitivity by RORγ has yet to be established (Mühlbauer et al., 2013). Further study is required to understand the modulation of lipid/glucose homeostasis and insulin sensitivity by RORγ.

## CONNECTION BETWEEN RORs, CIRCADIAN RHYTHM, AND METABOLIC SYNDROME

It has been well established that many behavioral and physiological activities display circadian rhythms that are regulated by endogenous clocks (Asher and Schibler, 2011; Bass, 2012; Mohawk et al., 2012). At the molecular level the clockwork consists of an integral network of several interlocking positive and negative transcriptional and translational feedback loops that include the transcriptional regulators brain and muscle ARNT-like 1 (Bmal1), neuronal PAS domain protein 2 (Npas2), circadian locomotor output cycles kaput (Clock), two cryptochrome proteins (Cry1, 2), the nuclear receptors Rev-erbα and -β, E4 promoter-binding protein 4 (E4bp4), and three period proteins (Per1-3).

Accumulating evidence suggests that disruption of circadian rhythm is closely associated with several pathologies, including sleep disorders, cancer and metabolic syndrome (Maury et al., 2010). Recent studies have established a strong link between the circadian clock machinery and the regulation of a number of metabolic pathways (Asher and Schibler, 2011; Bass, 2012). Bmal1, Clock, and Cry1 have been implicated in the regulation of glucose homeostasis and dysfunction in these proteins lead to impaired glucose tolerance (Rudic et al., 2004; Zhang et al., 2010). Hepatic overexpression of Cry1 has been reported to improve insulin-sensitivity in insulin-resistant *db/db* mice (Zhang et al., 2010). In addition, circadian oscillator components, such as Cry1, have been implicated in the regulation of immune responses (Castanon-Cervantes et al., 2010; Logan and Sarkar, 2012; Narasimamurthy et al., 2012). In *Cry1*^-/-^Cry2^-/-^ cells, NF-κB and protein kinase A (PKA) signaling pathways are constitutively activated resulting in elevated levels of circulating TNFα, Il-1β, and Il-6 (Narasimamurthy et al., 2012).

**FIGURE 2:**
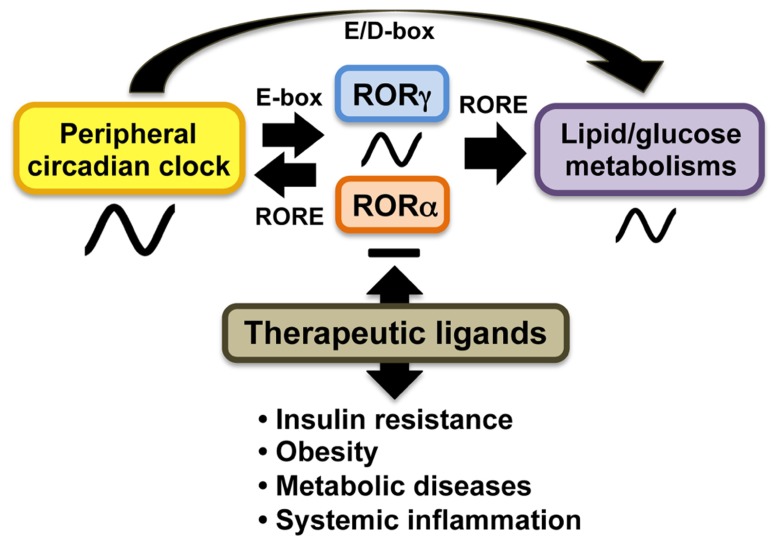
**In peripheral tissues, RORs function as intermediate regulators providing a link between clock proteins and their regulation of lipid and glucose metabolism.** The oscillatory pattern of expression of *RORγ* is regulated by circadian clock proteins Bmal1 and Clock or Npas2 through E-box, whereas RORα does not exhibit a strong oscillatory pattern of expression. In turn, RORγ activates expression of several clock genes through ROREs together with RORα. Both RORα and RORγ regulate the circadian pattern of expression of target genes involved in lipid/glucose metabolism. ROR (ant)agonists may modulate the amplitude of peripheral clock oscillation of these metabolic outputs and might be useful in the treatment of insulin resistance, obesity, and tissue inflammation.

A number of studies demonstrated that RORs play a role in the modulation of circadian behavior and clock gene expression ( Sato et al., 2004; Ueda et al., 2005; Duez and Staels, 2010; **Figure 2**). *Bmal1*, *Npas2*, *E4bp4*, and *Cry1* transcription are directly regulated by RORγ and RORα in several peripheral tissues through their interaction with ROREs in their regulatory regions (Crumbley et al., 2010; Takeda et al., 2011, 2012). RORγ1 appears to be the major ROR isotype modulating the circadian expression of clock genes in peripheral tissues. RORγ1 itself exhibits a strong oscillatory pattern of expression in several peripheral tissues, including kidney, liver, pancreas, and adipose tissue, while RORα exhibits only a weak circadian expression pattern (Mongrain et al., 2008; Takeda et al., 2012; Mühlbauer et al., 2013). The RORγ1 gene is directly regulated by Bmal1/Clock heterodimers which interact with two successive E-boxes in the RORγ1 promoter (Mongrain et al., 2008; Takeda et al., 2012). Recent studies have suggested that RORγ1 and RORα might provide a link between the clock machinery and their regulation of metabolic genes (Takeda et al., 2012; **Figure 2**). Data demonstrating that the circadian pattern of expression of a number of metabolic genes are regulated by clock proteins and RORs and observations showing that circadian expression of RORγ1 is controlled by the clock machinery suggested that RORs might function as downstream mediators in the mechanism by which clock proteins regulate the circadian expression of metabolic genes (Sato et al., 2004; Akashi and Takumi, 2005; Guillaumond et al., 2005; Ueda et al., 2005; Crumbley et al., 2010; Duez and Staels, 2010; Takeda et al., 2011, 2012). This is supported by observations showing that RORs regulate the circadian pattern of expression of a number of genes involved in the lipid/glucose homeostasis, including *Plin2*, sulfotransferase *Sul1E1*, the vasopressin receptor *Avpr1a*, and citrate synthase (*CS*), which exhibit roles in lipogenesis, glycogenolysis, and/or cholesterogenesis (Kang et al., 2007; Crumbley et al., 2012; Takeda et al., 2012). Thus, RORs appear to be part of the mechanism that links the circadian clock to its regulation of lipid/glucose homeostasis, inflammation, and insulin resistance (**Figure 2**).

## RORs AS THERAPEUTIC TARGETS FOR METABOLIC SYNDROME AND INSULIN RESISTANCE

X-ray crystallography studies of the LBD of RORα identified the presence of cholesterol in the ligand-binding pocket of RORα (Kallen et al., 2002). Subsequent studies identified cholesterol sulfate, 7-dehydrocholesterol, and 25-hydroxycholesterol as RORα agonists (Kallen et al., 2004). All-*trans* retinoic acid and the synthetic retinoid, ALRT 1550 were reported to bind and function as antagonists for RORβ and RORγ, but not RORα (Stehlin-Gaon et al., 2003). Recently, ursolic acid and several oxygenated sterols, including 7α-hydroxycholesterol (7α-OHC), 7β-hydroxycholesterol, 7-ketocholesterol, and 24S-hydroxycholesterol, were shown to function as inverse agonists to both RORα and RORγ (Wang et al., 2010a; Xu et al., 2011), while 20α-hydroxycholesterol and 22R-hydroxycholesterol acted as agonists (Jin et al., 2010). The LXR agonist T0901317 and several other synthetic derivatives, including SR1001, were identified as RORα and RORγ inverse agonists. Digoxin and several derivatives were identified as specific inhibitors for RORγ transcriptional activity (Fujita-Sato et al., 2011; Huh et al., 2011). The ROR (inverse) antagonists have been reported to repress the expression of ROR target genes and the activation of their promoter regulatory region by inhibiting the recruitment of co-activators. Moreover, ROR antagonists have been shown to inhibit Th17 cell differentiation and IL-17 production both *in vitro* and *in vivo* and to suppress the development of experimental autoimmune encephalomyelitis (Huh et al., 2011; Jetten, 2011; Solt et al., 2011). Therefore, antagonists for RORγ might be potential drugs for pharmacological intervention in the treatment and suppression of several autoimmune diseases, including multiple sclerosis, collagen-induced arthritis, rheumatoid arthritis, and asthma (Solt et al., 2010; Huh and Littman, 2012). Because of their role in regulating various features of metabolic syndrome, RORα and γ antagonists might also have beneficial effects in the management of obesity and insulin resistance.

## SUMMARY

The study of ROR-deficient mice has clearly demonstrated that RORα and RORγ are important in several physiological processes, including the regulation of several immune responses, lipid/glucose homeostasis, and circadian rhythm. These studies revealed that loss of RORα protects against the development of diet- and age-induced obesity, hepatosteatosis, glucose intolerance, and insulin resistance, while loss of RORγ protects against insulin resistance. These protective effects have been linked to suppression of the expression of multiple proinflammatory and metabolic genes. RORs regulate expression of some of these genes directly by binding ROREs in their regulatory region and in certain cases involves changes in their circadian pattern of expression. Although much progress has been made, what event or which ROR target genes are the primary driving force by which RORs influences WAT-associated inflammation, hepatosteatosis, and insulin resistance needs further study. With the increasing evidence for an interrelationship between the controls of lipid/glucose metabolism, inflammation and circadian rhythm, RORs might functions as intermediaries between the controls. With the discovery of ROR antagonists, RORs may provide a novel therapeutic target in the management of various aspect of metabolic syndrome.

## Conflict of Interest Statement

The authors declare that the research was conducted in the absence of any commercial or financial relationships that could be construed as a potential conflict of interest.
